# Abnormal developmental of structural covariance networks in young adults with heavy cannabis use: a 3-year follow-up study

**DOI:** 10.1038/s41398-024-02764-8

**Published:** 2024-01-20

**Authors:** Hui Xu, Jiahao Li, Huan Huang, Bo Yin, Dan-Dong Li

**Affiliations:** 1 School of Mental Health, Wenzhou Medical University, Wenzhou, 325035 China; 2 The Affiliated Kangning Hospital of Wenzhou Medical University, Zhejiang Provincial Clinical Research Center for Mental Disorder, Wenzhou, 325007 China; 3 Department of Neurosurgery, The Second Affiliated Hospital and Yuying Children’s Hospital of Wenzhou Medical University, Wenzhou, 325027 China; 4 Department of Neurology, The First Affiliated Hospital of Xi’an Jiaotong University, Xi’an, 710061 China; 5 Department of Radiology, The Second Affiliated Hospital and Yuying Children’s Hospital of Wenzhou Medical University, Wenzhou, 325027 China

**Keywords:** Neuroscience, Addiction, Predictive markers

## Abstract

Heavy cannabis use (HCU) exerts adverse effects on the brain. Structural covariance networks (SCNs) that illustrate coordinated regional maturation patterns are extensively employed to examine abnormalities in brain structure. Nevertheless, the unexplored aspect remains the developmental alterations of SCNs in young adults with HCU for three years, from the baseline (BL) to the 3-year follow-up (FU). These changes demonstrate dynamic development and hold potential as biomarkers. A total of 20 young adults with HCU and 22 matched controls were recruited. All participants underwent magnetic resonance imaging (MRI) scans at both the BL and FU and were evaluated using clinical measures. Both groups used cortical thickness (CT) and cortical surface area (CSA) to construct structural covariance matrices. Subsequently, global and nodal network measures of SCNs were computed based on these matrices. Regarding global network measures, the BL assessment revealed significant deviations in small-worldness and local efficiency of CT and CSA in young adults with HCU compared to controls. However, no significant differences between the two groups were observed at the FU evaluation. Young adults with HCU displayed changes in nodal network measures across various brain regions during the transition from BL to FU. These alterations included abnormal nodal degree, nodal efficiency, and nodal betweenness in widespread areas such as the entorhinal cortex, superior frontal gyrus, and parahippocampal cortex. These findings suggest that the topography of CT and CSA plays a role in the typical structural covariance topology of the brain. Furthermore, these results indicate the effect of HCU on the developmental changes of SCNs in young adults.

## Introduction

An increasing number of countries have legalized cannabis for medical or recreational purposes [1]. Recent reports indicate that approximately 209 million individuals worldwide are cannabis users, with the highest prevalence observed among young adults aged 18 to 25. Notably, during this developmental period, the brain undergoes reorganization of functional connections and experiences neurochemical changes that contribute to the promotion of adaptive behavioral regulation [2]. Heavy cannabis use (HCU) refers to the frequent and prolonged consumption of cannabis, leading to daily dysfunctional behavior [3]. Previous studies have provided evidence that long-term, high-level cannabis use can potentially increase susceptibility to neurotoxicity [4]. That may lead to structural and functional alterations in the brain, contributing to mental health and memory abnormalities. Conditions such as anxiety, depression, and memory impairment have been reported to be associated with HCU [4, 5]. HCU affects brain development in regions rich in cannabinoid-1 receptors (CB1), like the hippocampus, amygdala, cerebellum, cingulate cortex, and prefrontal cortex. These changes contribute to abnormalities in mental health and memory [6].

A previous longitudinal study revealed that young adults with HCU demonstrated a reduced growth rate of the right hippocampus. This structural alteration in the brain could potentially impact the local and global organization of brain structural networks [7]. Research findings indicate that individuals with HCU exhibit impaired working memory networks. Consequently, they require increased effort to perform working-memory tasks effectively [8]. Long-term cannabis use has been shown to significantly impact the adjustment and coordination of networks involved in self-awareness, such as the default mode network [9].

Furthermore, individuals with HCU exhibited an increased centrality in brain regions associated with sensory, motor, and attention networks. These alterations in network connectivity may serve as reliable neuroimaging markers for identifying HCU [10]. It is important to note that most existing studies have primarily focused on brain functional networks. At the same time, there remains a dearth of research investigating the developmental changes in brain structure using structural brain networks in young adults with HCU. Previous research has discovered that anatomical covariance structures enable network analysis similar to functional network analysis. Graph theoretical measures derived from anatomical covariance networks can exhibit alterations during development, aging, or disease, providing insights into the effects of environmental factors on the brain [11]. A fundamental hypothesis underlying this approach is that morphological correlations reflect the presence of axonal connections between different brain regions and are influenced by shared factors such as nutrition, genetics, and neurodevelopment [12]. Structure covariance networks (SCNs) specifically examine the covariant and coordinated patterns of gray matter morphology throughout the entire brain rather than focusing solely on individual regions or structures. This approach allows for a comprehensive exploration of how different brain regions interact and influence each other regarding their structural morphology [13]. Furthermore, SCNs have gained popularity in constructing group-level networks due to their lower computational intensity and reduced susceptibility to noise compared to functional networks. SCNs are advantageous for analyzing structural connectivity patterns in a group setting [14]. Multiple studies have identified specific abnormalities in the brain’s structural network linked to cannabis use [7, 15, 16]. Furthermore, previous studies have indicated that cannabis users exhibit distinct structural brain changes across different age cohorts [5].

Alterations in cortical thickness (CT) among cannabis users are associated with the severity of cannabis use and age of initiation. These associations can potentially be attributed to two factors: (i) deviations in neurodevelopmental trajectories and (ii) tissue loss or damage resulting from the neurotoxic effects of cannabis [17]. To comprehend neurodevelopmental changes in young adults with HCU, longitudinal studies must examine pre-existing differences and changes after cannabis initiation. However, few longitudinal studies have investigated the developmental changes in the integration and segregation of SCNs in young adults with HCU using cannabis for over three years. Further, based on graph theory, segregation refers to the degree to which a network’s elements form separate cliques or clusters, and integration refers to the capacity of the network as a whole to become interconnected and exchange information [18]. These two indicators can express the structural characteristics of networks from different perspectives [19]. In addition, previous research has found that both CT and cortical surface area (CSA) have distinct developmental trajectories and uncorrelated genetic backgrounds [20] and should be considered separate morphometric features in neurodevelopment [21, 22]. Hence, based on both CT and CSA, segregation and integration network graph theory indicators can help gain a deep understanding of the topography of CT and CSA in HCU.

This study employed CT and CSA to overcome these research gaps to construct SCNs. Subsequently, segregation and integration network graph theory indicators were calculated to explore the abnormal development of SCNs in young adults with HCU over three years, from baseline (BL) to the follow-up (FU) assessment. The primary goals of this study were twofold: (i) to discern developmental changes in global network measures of SCNs in young adults with HCU from BL to FU and (ii) to identify abnormal nodal network measures of SCNs in young adults with HCU. We hypothesized that (1) young adults with HCU showed significant deviations in small-worldness and local efficiency of CT and CSA compared to controls at baseline, while fewer differences were observed at the FU evaluation between groups. (2) Young adults with HCU displayed abnormal changes in nodal network measures across various brain regions, which were mainly distributed in addiction-related brain regions during the transition from BL to FU.

## Materials and methods

### Participants

For this study, we utilized data obtained from the OpenNEURO database (https://openneuro.org/datasets/ds000174/versions/1.0.1). Our analysis included 20 young adults with HCU and 22 carefully matched non-cannabis use controls. All participants underwent magnetic resonance imaging (MRI) scanning and clinical assessments at the study’s BL and FU stages, with an average interval duration of 39 ± 2.4 months. At the BL, HCU was defined as the consumption of cannabis for more than ten days per month consistently for a minimum of two years without seeking treatment or having a history of cannabis treatment [23]. The matched controls in this study were individuals who had used cannabis fewer than 30 times in their lifetime and had not used it within the past year [23]. For detailed information regarding the informed consent process with all participants, I recommend referring to the previous research conducted in this area [23].

### Clinical assessments

The severity of cannabis use was assessed using the Cannabis Use Disorder Identification Test (CUDIT) [24]. In addition, the severity of alcohol use was assessed using the Alcohol Use Disorder Identification Test (AUDIT) [25]. Additionally, the Mini International Neuropsychiatric Interview [26] was conducted by two experienced psychologists blinded to the study to assess the prevalence of mental disorders. For detailed information regarding participant characteristics and clinical evaluations, I recommend referring to the previous research where these details are outlined [23].

### MRI data acquisition

MRI scanning was conducted using a Philips Healthcare 3.0 T MRI scanner. Participants’ heads were secured in position using a custom-built head holder during the scanning process to ensure stability and accuracy. High-resolution structural images were acquired with the following parameters: echo time = 4.16 s, repetition time = 9.6 s, flip angle = 8°, slice thickness = 1.2 mm, field of view = 256 mm × 256 mm, matrix size = 256 × 256, voxel size = 1 × 1 × 1.2 mm^3^ and 182 slices.

### MRI data preprocessing and measurement of CSA and CT

Each participant’s structural T1-weighted MRI data were preprocessed using FreeSurfer v7.2.0 software package (http://surfer.nmr.mgh.harvard.edu). For detailed information regarding the surface-based morphology analysis, I recommend referring to the previous studies where the specifics of this analysis were documented [27–29]. The FreeSurfer pipeline processing involved several steps, including motion correction, removal of non-brain tissue, Talairach transformation, intensity normalization, gray/white matter boundary tessellation, topology correction, surface deformation, registration to a common spherical atlas, and cortical surface reconstruction. To obtain measurements of CT and CSA, the cortical morphologies were smoothed using a 10 mm full-width-at-half-maximum Gaussian kernel, following methodologies described in previous research [30–32]. CT was calculated at each vertex in the cortex by measuring the distance between the pial surface and the gray-white matter surface. This approach provides a local assessment of CT across the entire cortical surface. CSA was estimated by averaging the area of all faces connected to a specific vertex on the white matter surface. All outputs underwent meticulous inspection throughout the preprocessing phase, and manual corrections were applied as necessary. Subsequently, the average values of CT and CSA within 34 cortical parcellations were determined in each hemisphere and defined by the Desikan atlas [33], which has been used by recent research investigating brain SCNs [34, 35]. Each cortical region’s cortical surface indices, such as CT and CSA, were exported for subsequent analysis. In the following analyses, the potential confounding factors (including age, gender, CUDIT scores, AUDIT scores, and age at the onset of first cannabis use) were included as covariates.

### Construction of SCNs

We aimed to characterize the brain networks of young adults with HCU and controls by constructing structural covariance matrices. The statistical similarity between two brain regions was quantified using Pearson’s correlation coefficient, resulting in the construction of interregional correlation matrices (68 × 68) for each group at BL and FU. Group-level SCNs of CT and CSA were constructed separately for the two groups at the BL and FU stages. The values were transformed into *z*-scores using the Fisher transformation to enhance the normality of the correlation coefficients. The correlation matrix was then binarized using various sparsity thresholds, resulting in different percentages of connections. This process yielded a series of unweighted and undirected graphs for subsequent network analysis. To address the potential impact of threshold selection on small-world network parameters, we applied a wide range of sparsity thresholds (6–40%) to threshold the correlation matrices. This approach aimed to minimize uncertainty arising from threshold choice and ensure accurate estimation of small-world network architectures. It also helps to reduce the inclusion of spurious edges in each network, which is consistent with previous studies in the field [36, 37]. The lowest threshold was determined as the minimum network sparsity at which the resulting networks were fully connected, allowing for the estimation of small-worldness.

### Graph-based network analysis

**Fig. 1:**
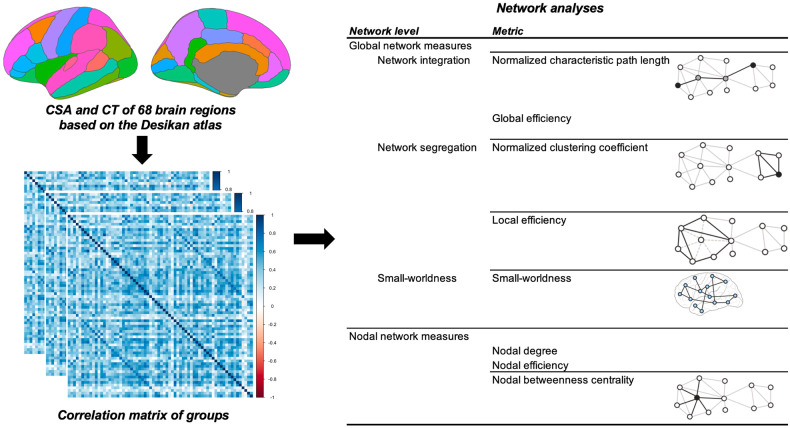
Schematic workflow of structural covariance network analyses in this study. CSA cortical surface area, CT cortical thickness.

Global and nodal network measures of the SCNs were calculated using the Brain Connectivity Toolbox [38] (Fig. 1). Standards of network integration, such as normalized characteristic path length and global efficiency, were computed. Measures of network segregation, including normalized clustering coefficient and local efficiency, were also determined. The small-worldness index was also computed, which indicates the balance between network integration and segregation. Nodal degree, nodal efficiency, and nodal betweenness centrality were examined to identify group differences in nodal network measures. For more details on these metrics, see Table S1 in the Supplementary materials section.

### Statistical analysis

A nonparametric permutation test was employed to investigate the statistical differences in network metrics between the HCU and control groups at BL and FU. Initially, network measures such as clustering, path length, efficiency, nodal efficiency, betweenness, and degree were computed separately for the HCU and control groups. Next, each subject’s CT or CSA values were randomly reassigned into two groups, maintaining the same sample size as the original groups. SCNs were recalculated for each of the two groups, and new values for the network metrics were obtained. This permutation process was repeated 1000 times, and statistical significance was determined if less than 5% of the between-group differences in the permutation distribution exceeded the observed between-group difference. To account for various densities, the area under the curve (AUC) was compared between the two groups at BL and FU, considering a density range of 0.06:0.01:0.4. *P*-value < 0.05 was statistically significant with false discovery rate (FDR) corrections after multiple comparisons. All figures for results were performed using R (Version 4.1.3; R Core Team, 2022) and RStudio (“Ghost Orchid” Release; RStudio Team, 2021), with the ggplot2 package (Version 3.4.4) and ggseg package (Version 1.6.5). Additionally, we also completed supplementary analyses to examine whether the topography of CT and CSA is altered in HCU at both BL and FU related to controls. Hence, an independent two-sample *t*-test was conducted to investigate group differences in CT and CSA of all brain regions.

## Results

### Participants and characteristics

No significant differences were found in age at BL (*t* (40) = −1.465, *P* = 0.151) and sex (*χ*^2^_(1)_ = 0.213, *P* = 0.645) between young adults with HCU and controls. Young adults with HCU demonstrated a significantly earlier age at the onset of first cannabis use compared to controls (*t* (40) = −4.367, *P* < 0.001), and the mean age at onset of frequent cannabis use for young adults with HCU was 16.20 ± 2.38 years old. However, there was no significant “group” × “time point” interaction effect on the score of CUDIT (*F*_(1, 80)_ = 0.033, *P* = 0.855) and score of AUDIT (*F*_(1, 80)_ = 0.082, *P* = 0.776). In the present study, none of the participants exhibited a prevalence of mental disorders. The demographic information of all participants is depicted in Table S2.

### Group differences of global network integration and segregation measures based on CSA

**Fig. 2:**
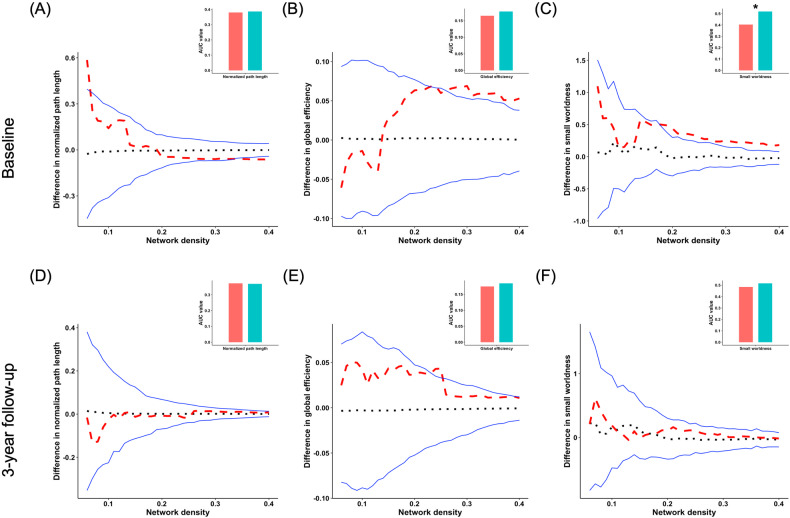
Group differences of integration and small-worldness of CSA. Group differences in “integration” and “small-worldness” metrics of structural covariance networks based on the cortical surface area at baseline and 3-year follow-up at the range of 6–40% network sparsity, including **A**, **D** normalized path length, **B**, **E** global efficiency, and **C**, **F** “small-worldness”. The upper and lower blue lines represented a 95% confidence interval, whereas the black dot line in the middle denoted the mean difference after 1000 permutations. The red line represents the true group differences, which fall outside the confidence interval, indicating significant group differences (*P* < 0.05) under the current threshold. The positive values indicate young adults with HCU > HCs, and the negative values indicate young adults with HCU < HCs. The subpanels showed group differences in the area under the curve (AUC) value in each metric of SCNs. Compared with HCs, the young adults with HCU showed significantly higher AUC value of small worldness at baseline. *P < 0.05.

Young adults with HCU exhibited significantly altered small-worldness measures compared to controls at BL. However, there were no significant differences between young adults with HCU and controls at FU. Moreover, no significant group differences were found in global network integration measures at both BL and FU (Fig. 2).

**Fig. 3:**
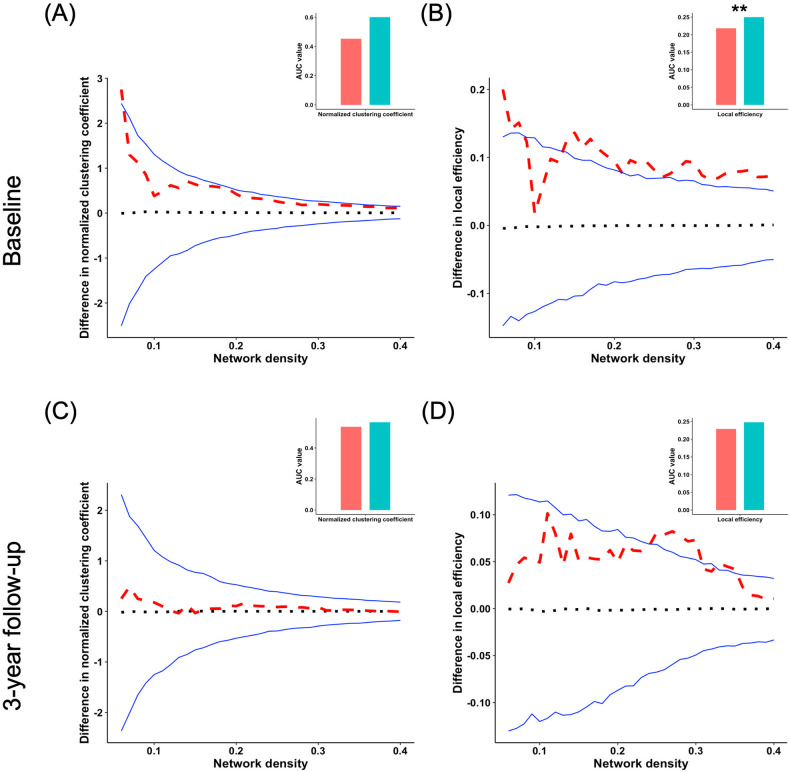
Group differences of segregration of CSA. Group differences in “segregation” metrics of structural covariance networks based on the cortical surface area at baseline and 3-year follow-up at the range of 6–40% network sparsity, including **A**, **C** normalized clustering coefficient, and **B**, **D** local efficiency. The upper and lower blue lines represented a 95% confidence interval, whereas the black dot line in the middle denoted the mean difference after 1000 permutations. The red line represents the true group differences, which fall outside the confidence interval and indicate significant group differences (*P* < 0.05) under the current threshold. The positive values indicate young adults with HCU > HCs, and the negative values indicate young adults with HCU < HCs. The subpanels showed group differences in the area under the curve (AUC) value in each metric of SCNs. Compared with HCs, the young adults with HCU showed significantly higher AUC value of local efficiency at baseline. **P < 0.01.

Additionally, at BL, young adults with HCU demonstrated abnormal local efficiency compared to controls. However, at FU, the two groups had no significant difference in local efficiency (Fig. 3). No significant group differences were found in other global network segregation measures at both BL and FU. The *P*-values for these metrics are listed in Table 1. Further, independent two-sample *t*-tests found that no significant group differences in both CT and CSA of all brain regions were observed (Table S3).

**Table 1:** Results of permutation tests for differences in the integration and segregation measures of SCNs between groups at BL and FU (*P*-value after FDR correction).

	Timepoint	Integration measures		Small-worldness	Segregation measures	
		Normalized path length	Global efficiency		Normalized clustering coefficient	Local efficiency
CSA	BL	0.736	0.194	0.05	0.146	0.002
	FU	0.768	0.157	0.648	0.778	0.053
CT	BL	0.077	0.192	0.006	0.092	0.004
	FU	0.854	0.852	0.627	0.759	0.659

*SCNs* structural covariance networks, *CSA* cortical surface area, *CT* cortical thickness, *BL* baseline, *FU* 3-year follow-up, *FDR* false discovery rate.

### Group differences of global network integration and segregation measures based on CT

**Fig. 4:**
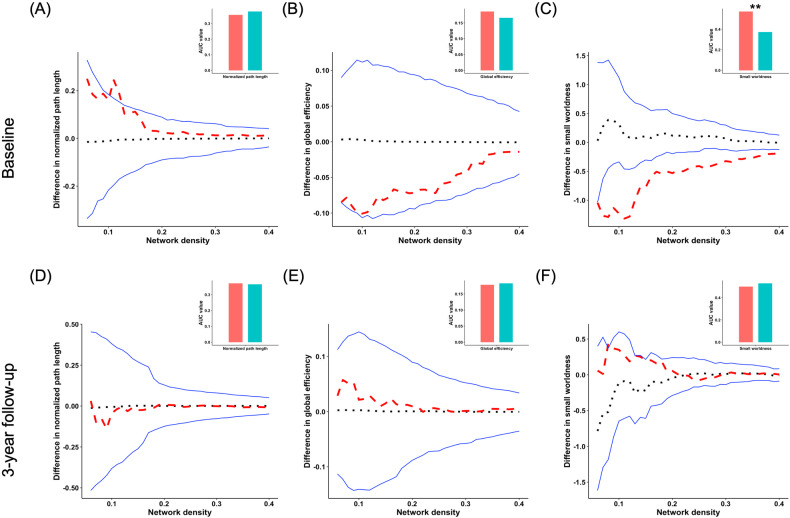
Group differences of integration and small-worldness of CT. Group differences in “integration” and “small-worldness” metrics of structural covariance networks based on cortical thickness at baseline and 3-year follow-up at the range of 6–40% network sparsity, including **A**, **D** normalized path length, **B**, **E** global efficiency, and **C**, **F** “small-worldness”. The upper and lower blue lines represented a 95% confidence interval, whereas the black dot line in the middle denoted the mean difference after 1000 permutations. The red line represents the true group differences, which fall outside the confidence interval and indicate significant group differences (*P* < 0.05) under the current threshold. The positive values indicate young adults with HCU > HCs, and the negative values indicate young adults with HCU < HCs. The subpanels showed group differences in the area under the curve (AUC) value in each metric of SCNs. Compared with HCs, the young adults with HCU showed significantly lower AUC value of small worldness at baseline. **P < 0.01.

While young adults with HCU exhibited significant alterations in small-worldness measures compared to controls at BL, no significant difference was observed between young adults with HCU and controls at FU. However, no significant group differences were found in global network integration measures at both BL and FU (Fig. 4).

**Fig. 5:**
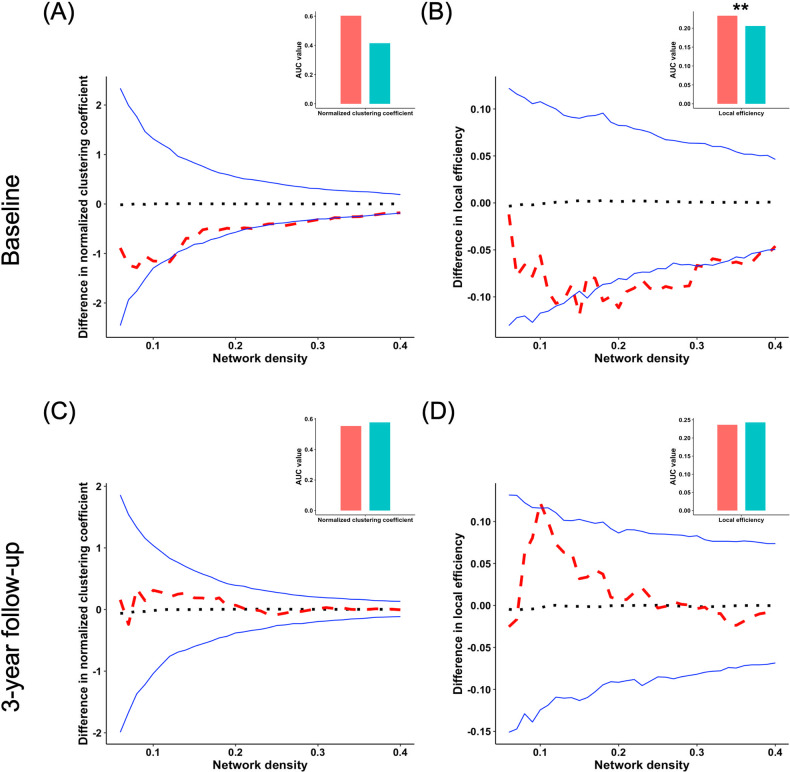
Group differences of segregration of CT. Group differences in “segregation” metrics of structural covariance networks based on cortical thickness at baseline and 3-year follow-up at the range of 6%-40% network sparsity, including **A**, **C** normalized clustering coefficient, and **B**, **D** local efficiency. The upper and lower blue lines represented a 95% confidence interval, whereas the black dot line in the middle denoted the mean difference after 1000 permutations. The red line represents the true group differences, which fall outside the confidence interval and indicate significant group differences (*P* < 0.05) under the current threshold. The positive values indicate young adults with HCU > HCs, and the negative values indicate young adults with HCU < HCs. The subpanels showed group differences in the area under the curve (AUC) value in each metric of SCNs. Compared with HCs, the young adults with HCU showed significantly lower AUC value of local efficiency at baseline. **P < 0.01.

Furthermore, at BL, young adults with HCU displayed abnormal local efficiency compared to controls. However, at FU, the two groups had no significant difference in local efficiency (Fig. 5). Other global network segregation measures at BL and FU showed no significant group differences. The *P*-values for these metrics are provided in Table 1.

### Group differences of nodal network measures of CSA and CT

**Fig. 6:**
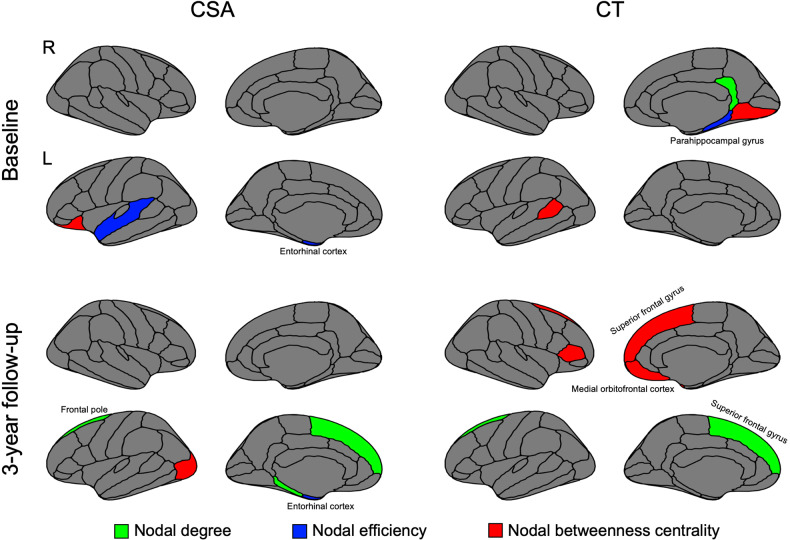
Group differences in nodal network metrics (nodal degree, nodal efficiency, and nodal betweenness centrality) of structural covariance networks based on cortical surface area and cortical thickness at baseline and 3-year follow-up. Regions that showed significant differences in AUC in the range from 6% to 40% network sparsity in nodal degree, nodal efficiency, and nodal betweenness centrality between groups were colored (*P* < 0.05, false discovery rate corrected). The green color represented regions that have altered nodal degrees in young adults with HCU. The blue color denoted regions that have altered nodal efficiency in young adults with HCU. The red color represented regions that have altered nodal betweenness centrality in young adults with HCU. This graph was plotted using the R and RStudio with both ggplot2 and ggseg packages.

According to permutation tests, significant group differences were observed in nodal network measures, including nodal degree, nodal efficiency, and nodal betweenness centrality at both BL and FU. The corresponding statistical results are presented in Fig. 6 and Table S4.

Regarding CSA, at BL, young adults with HCU exhibited significant alterations in nodal degree within the left entorhinal cortex and superior temporal gyrus compared to controls. Additionally, abnormal nodal efficiency was observed in the left entorhinal cortex, while altered nodal betweenness centrality was found in the left lateral orbitofrontal cortex (OFC). These findings suggest specific disruptions in brain network properties associated with CSA in young adults with HCU. Compared to controls, young adults with HCU continued to show abnormal nodal degrees in the left entorhinal cortex (EC) at FU. Additionally, altered nodal efficiency was observed in widespread regions, including the left EC, parahippocampal cortex (PHC), superior frontal gyrus (SFG), frontal pole, and temporal pole. These findings indicate persistent disruptions in nodal network measures in multiple brain regions associated with HCU in young adults.

Regarding CT, at BL, young adults with HCU showed altered nodal degree in the right parahippocampal gyrus and abnormal nodal efficiency in the right isthmus cingulate. They changed nodal betweenness centrality in the left banks of the superior temporal sulcus and the right lingual gyrus. At FU, compared to controls, young adults with HCU exhibited abnormal nodal efficiency in the left SFG and altered nodal betweenness centrality in bilateral SFG and right medial OFC. These findings indicate specific disruptions in nodal network measures associated with CT in young adults with HCU.

## Discussion

This study is the first to investigate the abnormal development of SCNs in young adults with HCU, considering both CT and CSA. The findings revealed that at BL, young adults with HCU exhibited significant alterations in small-worldness and abnormal local efficiency in CT and CSA compared to controls. However, no significant differences were observed between groups at FU. Additionally, nodal network measures showed abnormal development in widespread brain regions, including the entorhinal cortex (EC), parahippocampal cortex (PHC), superior frontal gyrus (SFG), and orbitofrontal cortex (OFC). These findings shed light on the impact of HCU on the brain’s structural organization in young adults and the involvement of key brain regions in various functional networks.

Previous research has demonstrated that small-worldness reflects a balanced network state, combining network integration and segregation. It is characterized by a high clustering coefficient and short average path length, enabling efficient information processing with minimal connectivity costs [38]. Local efficiency measures network segregation, representing the average of the inverse shortest path lengths between neighboring nodes. It quantifies the efficiency of information transfer within the local neighborhoods of a node [38]. This study observed altered small-worldness and local efficiency in CT and CSA of young adults with HCU at BL, consistent with previous DTI-based graph theory findings [39]. These findings suggest that in individuals with HCU, white matter fiber tracts undergo changes [33] and alterations in structural brain regions. These changes ultimately contribute to abnormal global network metrics of the structural brain. However, it is essential to note that at FU, no significant group differences were observed. This implies that at BL, the architecture of SCNs exhibits abnormalities, resulting in disrupted information transfer among neighboring nodes. Aberrant alterations in SCNs may gradually be compensated through continuous reconfiguration of the networks; this may be independent and unaffected by cannabis use. In addition, we found different network anomalies at BL and FU, and we think that it may be the transformation of the abnormal regions that completes the network function compensation; it was a dynamic development that will complement the findings of the previous cross-sectional study [39, 40]. While no significant modifications in characteristic path length, clustering coefficient, and global efficiency were observed at both BL and FU, altered small-worldness in young adults with HCU suggests impaired network efficiency and information processing in this population [36].

In terms of nodal network measures, young adults with HCU at BL displayed abnormal nodal degrees in the EC and PHC, both of which are implicated in the memory network [41] and default mode network (DMN) [42]. The EC is a hub region in the memory network, acting as the gateway for memory-related information. It selectively regulates connections with the hippocampus and prefrontal cortex, facilitating efficient memory processing and retrieval [41]. In a previous study, CT alterations in the EC were linked to genetic abnormalities in cannabis addiction. Additionally, these CT changes were associated with the abundance of CB1 in the EC, suggesting a relationship between structural alterations, genetics, and cannabis addiction [43, 44]. Long-term cannabis use is linked to impaired memory function, which predicts the severity of cannabis addiction, indicating a relationship between memory impairment and addictive behaviors [45, 46]. The PHC assists the EC in transmitting information to the hippocampus [41]; abnormal connectivity of the PHC can influence memory function by disrupting the connection between the DMN and the memory network [47]. Previous research has shown that chronic cannabis use in young adults is associated with weakened functional connectivity (FC) within the DMN and enhanced FC between the DMN and other networks [48]. In addition, changes in gray matter volume within the DMN are associated with alterations in the cerebellar network, which are associated with early cannabis use and cannabis-induced psychiatric disorders [49]. As a result, cannabis may affect the structure or functionality of the DMN, which in turn affects other networks and produces memory and psychiatric disorders [49]. Furthermore, the abnormalities observed in nodal network measures of brain regions involved in the memory network and DMN persisted at FU, with an even broader range of anomalies. These findings suggest that alterations in the memory network may be prolonged and fixed, while DMN abnormalities may play a significant role in affecting the structure of the memory network.

Interestingly, at both BL and FU, abnormalities in nodal indicators were observed in the SFG, temporal pole, and OFC of young adults with HCU. These regions are associated with the frontoparietal, salience, and reward networks, respectively [42, 50–52]. The frontoparietal network performs executive functions, such as IQ, verbal learning, and memory [53]. The SFG is responsible for motor tasks and working memory in the frontoparietal network, as well as being a region where the DMN and frontoparietal network overlap [42]. Studies on the function of SFG subregions have shown that three major subregions of the SFG have anatomically and functional connections with motor and cognition-related regions, the anteromedial SFG has close connections with the anterior cingulate cortices (ACC), and the posterior SFG has connections with amygdala and thalamus, which are also critical regions for addictive behaviors [54]. Cannabis addiction reduces metabolic levels in the frontal lobe, which causes dysfunction in the frontal cortex and decreased functional connectivity with the ACC [55]. Cannabis also affects the structure and function of the amygdala, resulting in abnormalities in the reward process, including amygdala, ACC, and frontal lobe [56]. Our study found that abnormalities in the nodal indicators of the SFG persisted at BL and FU, suggesting that the SFG is abnormally long-lasting in its association with other addiction-related regions during cannabis addiction and may undergo similar or opposite structural changes with them; previous research found increased CT in the frontoparietal region of HCU, associated with impaired intelligence [43]. From the network’s perspective, SFG is a vital connection between the frontoparietal network and the DMN, facilitating their interactions during attention and self-referential processing [54]. Our findings indicated that frontoparietal network abnormalities were more extensive and prominent at FU than at BL, which suggested that the effects of cannabis on the frontoparietal network were dynamically changing and might be mediated through abnormalities in the DMN.

Additionally, the salience network, which plays a crucial role in modulating attention, emotion, and behavioral responses, is a critical mediator in the interaction between perception and emotion [57]. The temporal pole consists of three subregions that serve as intermediary nodes connecting the salience network to the social-emotional network, frontoparietal network, and DMN [50, 58, 59]. Among them, the temporal pole is linked to some addiction-related regions, such as orbitofrontal cortex, amygdala, and ACC [50]. Studies have shown that cannabis significantly reduces the volume and thickness of the temporal pole, ACC, and amygdala, and this change has been associated with addiction-induced depressive states [60]. In addition, cannabis also increases functional connectivity between the temporal lobe and the ACC; such abnormal connectivity is associated with anxiety state [61]. The observed anomalies in nodal indicators of the temporal pole suggest that abnormalities in the salience network can impact the development of other cognitive-related networks, subsequently influencing various cognitive functions. Additionally, understanding the abnormal growth of the salience network is crucial in the context of neuropsychiatric disorders, including addiction and other related conditions [62]. While studies on the salience network in individuals with HCU are limited, our findings highlight the potential of salience network abnormalities in further understanding addiction symptoms and abnormal mental states in HCU.

The reward network is essential for reward processing, motivation, and addiction. It is vital in perceiving, evaluating, and predicting rewarding stimuli, shaping behavior, and contributing to addictive processes [51, 63]. The OFC is involved in goal-driven reward tasks and has a dual impact on addictive behavior through inhibitory and excitatory regulation mechanisms [51]. The OFC responds more significantly to the BOLD in cannabis reward, and its abnormally increased activity is also associated with cannabis-related negative mood [61]. Furthermore, the abnormal connectivity of the OFC to the striatum and amygdala persisted after cannabis withdrawal [64]; adolescent cannabis use is associated with reduced thickness in the OFC. However, these changes in the OFC are not found to be associated with the dose and duration of cannabis use [65]. Our study showed the nodal indicators of the OFC at BL and FU were consistently abnormal, suggesting that abnormalities in the OFC were associated with cannabis addiction formation and negative mood [64]. Moreover, OFC also acts as a bridge between the DMN and limbic areas, facilitating the integration of cognitive and emotional processes and regulating self-referential processing and emotional responses [66]. Thus, cannabis addiction-induced anomalies in the reward network may be long-term and permanent and may also impact the DMN via OFC.

The results suggest that structural alterations in the memory network of individuals with HCU persist over time. Additionally, the abnormalities in the DMN may progressively increase with prolonged cannabis use, potentially impacting other networks involved in executive, emotional, and reward processes. The study demonstrated that the structural covariance network approach could capture abnormal developmental patterns in regions such as the frontal lobe, providing valuable insights into addiction mechanisms and potential risks associated with HCU.

The present study has some limitations that should be acknowledged. Firstly, the small sample sizes of both groups may limit the generalizability of the findings. Future studies with larger sample sizes are needed to validate the results. Secondly, the follow-up period of 3 years may not capture long-term changes in SCNs. Future studies with longer follow-up durations are necessary to understand the trajectory of SCNs over time in individuals with HCU. Thirdly, due to graph-based network analysis, this study can’t correlate global and nodal network measures of the SCNs with clinical assessments, including CUDIT and AUDIT scores. Future research should take the relationships between alterations of SCNs and addiction behavior assessments into account. Further, our findings might reflect the abnormal development of structural covariance networks associated with HCU in young adults, and a conclusion about causality can’t be made in this study. Future research should be conducted to investigate whether HCUs can influence SCNs or vice versa.

In summary, young adults with HCU displayed significant alterations in small-worldness and local efficiency of both CT and CSA at BL. Further, HCU individuals exhibited abnormal nodal measures such as degree, efficiency, and betweenness in various brain regions across the FU. These findings highlight the impact of HCU on the developmental changes of SCNs in young adults and suggest that CT and CSA play a role in the brain’s structural topology.

### Supplementary information

Supplementary Materials

## Data Availability

The data that support the findings of this study are available on request from the corresponding authors.
