# Supplementary material for: Abnormal developmental of structural covariance networks in young adults with heavy cannabis use: a 3-year follow-up study
Source: Transl Psychiatry. 2024 Jan 20;14:45. doi: 10.1038/s41398-024-02764-8 (PMC10799944; doi:10.1038/s41398-024-02764-8)
Supplement: Supplementary file 1 — Supplementary Materials [file 41398_2024_2764_MOESM1_ESM.docx]

**Supplementary materials**

| **Table S1.** Detailed information of each network metric in structural covariance networks | | | | |
| --- | --- | --- | --- | --- |
| Network level | | Metric | Definition | Interpretation |
| Global network measures | | | | |
|  | Integration | Normalized characteristic path length | The ratio between the shortest path length of actual and random graphs |  |
|  |  | Global efficiency | The inverse of the harmonic mean of the minimum path length between each pair of nodes | A measure of parallel information transformation |
|  | Segregation | Normalized clustering coefficient | The ratio between the clustering coefficients of actual and random graph | The extent of local information communication of the network |
|  |  | Local efficiency | The averaged local efficiency of each node, which is defined as the global efficiency of sub-graph of the node | A measure of fault tolerance of the network, indicating how well each sub-graph exchanges information when the index node is eliminated |
|  | Worldness | Small-worldness | Ratio of standardized clustering coefficient to standardized path length | Extent of local clustering among nodes within a network. Higher values indicative of highly specialized and segregated regions for functional specialization that are strongly and efficiently connected for integration. |
| Nodal network measures | | | | |
|  |  | Nodal degree | The sum of the edges that connected to the node | Network sparsity or density of connectivity among brain regions within the whole network. |
|  |  | Nodal efficiency | The inverse of the harmonic mean of the minimum path length between the node and all other nodes | The information propagation ability of a node with the rest of the nodes in the network |
|  |  | Nodal betweenness centrality | The fraction of all shortest paths in the network that pass through the node | The influence of a node over information flow between all other nodes in the network |

| **Table S2**. Demographic information of young adults with HCU and Controls. | | | | | | |
| --- | --- | --- | --- | --- | --- | --- |
|  | Young adults with HCU | | Controls | | *t/χ^2^*-value | *P*-value |
| Age at baseline (years) | 20.53(2.11) | | 21.56(2.45) | | -1.465 | 0.151 |
| Gender (F/M) | 5/15 | | 8/14 | | 0.213 | 0.645 |
| Age at onset first cannabis use (years) | 14.50(1.65) | | 18.46(2.99) | | -4.367 | 0.001 |
| Age at onset frequent cannabis use (years) | 16.20(2.38) | | NA | | NA | NA |
| Time point | BL | FU | BL | FU | *F*-value | *P*-value |
| Score of CUDIT | 12.70(6.59) | 13.25(8.31) | 0.05(0.21) | 0.18(0.39) | 0.033 | 0.855 |
| Score of AUDIT | 6.25(3.35) | 8.50(4.96) | 4.41(3.38) | 6.18(3.45) | 0.082 | 0.776 |
| *Notes*. HCU, heavy cannabis use; CUDIT, Cannabis Use Disorder Identification Test; AUDIT, Alcohol Use Disorder Identification Test; NA, Not Available; BL, baseline; FU, follow-up. Data was presented as Mean (SD) unless otherwise stated | | | | | | |

| **Table S3**. Group differences in CSA and CT of all brain regions at baseline and 3-year follow up | | | | | | | |
| --- | --- | --- | --- | --- | --- | --- | --- |
| CSA | | | | | | | |
|  | Baseline | | |  | 3-year follow-up | | |
| Brain regions | T-value | P-value | Effect size |  | T-value | P-value | Effect size |
| L_Banks of the superior temporal sulcus | 0.856 | 0.397 | 0.263 |  | 0.048 | 0.962 | 0.015 |
| L_Caudal anterior cingulate | 1.929 | 0.061 | 0.595 |  | 1.255 | 0.217 | 0.384 |
| L_Caudal middle frontal | 0.082 | 0.935 | 0.026 |  | -0.426 | 0.672 | -0.132 |
| L_Cuneus | 0.637 | 0.528 | 0.199 |  | 0.325 | 0.747 | 0.101 |
| L_Entorhinal cortex | 1.187 | 0.055 | 0.378 |  | 1.320 | 0.194 | 0.408 |
| L_Fusiform gyrus | 0.999 | 0.324 | 0.309 |  | 0.780 | 0.440 | 0.241 |
| L_Inferior parietal lobule | 0.449 | 0.656 | 0.139 |  | 0.224 | 0.824 | 0.069 |
| L_Inferior temporal gyrus | 1.402 | 0.170 | 0.440 |  | 1.414 | 0.165 | 0.434 |
| L_Isthmus of the cingulate | 0.473 | 0.639 | 0.144 |  | 0.394 | 0.696 | 0.121 |
| L_Lateral occipital gyrus | 1.443 | 0.157 | 0.449 |  | 1.144 | 0.260 | 0.356 |
| L_Lateral orbitofrontal cortex | 1.272 | 0.211 | 0.395 |  | -0.144 | 0.886 | -0.044 |
| L_Lingual gyrus | 1.252 | 0.218 | 0.390 |  | 1.191 | 0.241 | 0.371 |
| L_Medial orbitofrontal cortex | 1.059 | 0.296 | 0.329 |  | -0.064 | 0.950 | -0.020 |
| L_Middle temporal gyrus | 1.701 | 0.097 | 0.526 |  | 0.974 | 0.336 | 0.297 |
| L_Parahippocampal gyrus | -0.006 | 0.995 | -0.002 |  | 0.270 | 0.789 | 0.084 |
| L_Paracentral gyrus | 1.149 | 0.138 | 0.255 |  | -0.131 | 0.897 | -0.040 |
| L_Pars opercularis | 0.116 | 0.908 | 0.036 |  | 0.548 | 0.587 | 0.170 |
| L_Pars orbitalis | 0.742 | 0.463 | 0.232 |  | -0.066 | 0.948 | -0.020 |
| L_Pars triangularis | 0.010 | 0.992 | 0.003 |  | 0.100 | 0.921 | 0.031 |
| L_Pericalcarine fissure | 1.118 | 0.271 | 0.338 |  | -0.277 | 0.783 | -0.085 |
| L_Postcentral gyrus | 0.542 | 0.591 | 0.168 |  | -1.078 | 0.288 | -0.328 |
| L_Posterior cingulate | 0.886 | 0.381 | 0.268 |  | -0.389 | 0.700 | -0.118 |
| L_Precentral gyrus | 0.600 | 0.552 | 0.183 |  | -0.111 | 0.912 | -0.034 |
| L_Precuneus | -0.041 | 0.967 | -0.013 |  | -0.563 | 0.577 | -0.177 |
| L_Rostral anterior cingulate | 0.099 | 0.922 | 0.031 |  | -0.201 | 0.842 | -0.062 |
| L_Rostral middle frontal gyrus | 1.339 | 0.126 | 0.237 |  | 1.189 | 0.235 | 0.177 |
| L_Superior frontal gyrus | 1.184 | 0.244 | 0.368 |  | 0.849 | 0.401 | 0.265 |
| L_Superior parietal lobule | 1.702 | 0.096 | 0.522 |  | 1.223 | 0.229 | 0.380 |
| L_Superior temporal gyrus | 1.373 | 0.178 | 0.416 |  | 1.201 | 0.237 | 0.365 |
| L_Supramarginal gyrus | 0.085 | 0.933 | 0.026 |  | -0.463 | 0.646 | -0.142 |
| L_Frontal pole | 1.488 | 0.145 | 0.459 |  | 0.240 | 0.812 | 0.076 |
| L_Temporal pole | 1.200 | 0.123 | 0.195 |  | 0.617 | 0.541 | 0.192 |
| L_Transverse temporal cortex | 0.808 | 0.424 | 0.245 |  | 1.384 | 0.174 | 0.425 |
| L_Insula | 0.733 | 0.468 | 0.224 |  | 0.524 | 0.603 | 0.162 |
| R_Banks of the superior temporal sulcus | 0.985 | 0.331 | 0.308 |  | 0.114 | 0.910 | 0.035 |
| R_Caudal anterior cingulate | 1.031 | 0.310 | 0.323 |  | 0.256 | 0.799 | 0.080 |
| R_Caudal middle frontal | 0.739 | 0.464 | 0.230 |  | 0.238 | 0.813 | 0.075 |
| R_Cuneus | -0.095 | 0.925 | -0.029 |  | 0.018 | 0.986 | 0.006 |
| R_Entorhinal cortex | 1.565 | 0.125 | 0.480 |  | 0.015 | 0.988 | 0.005 |
| R_Fusiform gyrus | 1.249 | 0.219 | 0.384 |  | 0.293 | 0.771 | 0.088 |
| R_Inferior parietal lobule | 0.358 | 0.723 | 0.112 |  | -0.239 | 0.812 | -0.073 |
| R_Inferior temporal gyrus | 1.936 | 0.061 | 0.606 |  | 1.071 | 0.291 | 0.326 |
| R_Isthmus of the cingulate | 0.988 | 0.329 | 0.301 |  | -0.075 | 0.940 | -0.023 |
| R_Lateral occipital gyrus | 0.662 | 0.512 | 0.203 |  | 0.677 | 0.502 | 0.209 |
| R_Lateral orbitofrontal cortex | 1.011 | 0.318 | 0.313 |  | 0.602 | 0.551 | 0.185 |
| R_Lingual gyrus | 1.063 | 0.294 | 0.326 |  | 0.029 | 0.977 | 0.009 |
| R_Medial orbitofrontal cortex | 1.103 | 0.277 | 0.342 |  | 0.999 | 0.324 | 0.307 |
| R_Middle temporal gyrus | 1.120 | 0.270 | 0.349 |  | 0.556 | 0.582 | 0.170 |
| R_Parahippocampal gyrus | 0.085 | 0.933 | 0.027 |  | -1.469 | 0.150 | -0.445 |
| R_Paracentral gyrus | 0.634 | 0.530 | 0.195 |  | -0.258 | 0.798 | -0.081 |
| R_Pars opercularis | 0.681 | 0.501 | 0.205 |  | 0.027 | 0.978 | 0.008 |
| R_Pars orbitalis | 1.144 | 0.260 | 0.356 |  | 0.570 | 0.572 | 0.177 |
| R_Pars triangularis | 0.759 | 0.452 | 0.232 |  | 0.164 | 0.870 | 0.050 |
| R_Pericalcarine fissure | 0.478 | 0.635 | 0.147 |  | -0.040 | 0.968 | -0.012 |
| R_Postcentral gyrus | -0.529 | 0.600 | -0.166 |  | -1.221 | 0.230 | -0.382 |
| R_Posterior cingulate | 0.382 | 0.704 | 0.117 |  | -0.210 | 0.835 | -0.065 |
| R_Precentral gyrus | 0.767 | 0.448 | 0.238 |  | -0.458 | 0.649 | -0.143 |
| R_Precuneus | 0.570 | 0.572 | 0.177 |  | 0.364 | 0.718 | 0.114 |
| R_Rostral anterior cingulate | 0.819 | 0.419 | 0.259 |  | 0.070 | 0.944 | 0.022 |
| R_Rostral middle frontal gyrus | 1.115 | 0.061 | 0.149 |  | 1.099 | 0.279 | 0.334 |
| R_Superior frontal gyrus | 1.370 | 0.179 | 0.426 |  | 0.565 | 0.575 | 0.175 |
| R_Superior parietal lobule | 1.385 | 0.174 | 0.424 |  | 0.754 | 0.455 | 0.234 |
| R_Superior temporal gyrus | 1.275 | 0.210 | 0.396 |  | -0.027 | 0.979 | -0.008 |
| R_Supramarginal gyrus | 0.159 | 0.874 | 0.050 |  | -0.303 | 0.764 | -0.095 |
| R_Frontal pole | -0.703 | 0.487 | -0.220 |  | 0.710 | 0.482 | 0.220 |
| R_Temporal pole | 1.897 | 0.065 | 0.587 |  | 1.508 | 0.139 | 0.465 |
| R_Transverse temporal cortex | 0.619 | 0.540 | 0.190 |  | 1.016 | 0.316 | 0.308 |
| R_Insula | 0.499 | 0.620 | 0.154 |  | -0.825 | 0.414 | -0.256 |
| CT | | | | | | | |
| Brain regions | Baseline | | |  | 3-year follow-up | | |
| L_Banks of the superior temporal sulcus | 0.659 | 0.515 | 0.207 |  | 0.796 | 0.431 | 0.247 |
| L_Caudal anterior cingulate | 0.705 | 0.486 | 0.222 |  | 1.443 | 0.157 | 0.442 |
| L_Caudal middle frontal | 1.566 | 0.126 | 0.488 |  | 0.307 | 0.761 | 0.094 |
| L_Cuneus | 0.440 | 0.662 | 0.136 |  | -0.023 | 0.982 | -0.007 |
| L_Entorhinal cortex | -0.012 | 0.991 | -0.004 |  | 0.345 | 0.732 | 0.107 |
| L_Fusiform gyrus | 0.815 | 0.421 | 0.255 |  | 0.806 | 0.425 | 0.249 |
| L_Inferior parietal lobule | 0.353 | 0.726 | 0.110 |  | 1.509 | 0.140 | 0.459 |
| L_Inferior temporal gyrus | 0.564 | 0.577 | 0.178 |  | 0.035 | 0.972 | 0.011 |
| L_Isthmus of the cingulate | 1.060 | 0.296 | 0.324 |  | 0.893 | 0.378 | 0.278 |
| L_Lateral occipital gyrus | 0.090 | 0.929 | 0.028 |  | -0.800 | 0.428 | -0.246 |
| L_Lateral orbitofrontal cortex | -1.021 | 0.314 | -0.318 |  | 1.933 | 0.062 | 0.583 |
| L_Lingual gyrus | 0.424 | 0.674 | 0.131 |  | 0.401 | 0.690 | 0.125 |
| L_Medial orbitofrontal cortex | -0.162 | 0.872 | -0.050 |  | 1.280 | 0.228 | 0.110 |
| L_Middle temporal gyrus | -0.725 | 0.473 | -0.226 |  | -0.475 | 0.637 | -0.148 |
| L_Parahippocampal gyrus | 1.120 | 0.062 | 0.171 |  | 0.280 | 0.781 | 0.088 |
| L_Paracentral gyrus | 0.210 | 0.835 | 0.065 |  | 1.031 | 0.309 | 0.319 |
| L_Pars opercularis | 1.320 | 0.195 | 0.402 |  | 1.548 | 0.130 | 0.481 |
| L_Pars orbitalis | 0.451 | 0.654 | 0.139 |  | 1.362 | 0.181 | 0.421 |
| L_Pars triangularis | 1.015 | 0.316 | 0.312 |  | 1.999 | 0.053 | 0.625 |
| L_Pericalcarine fissure | 0.618 | 0.540 | 0.191 |  | -0.027 | 0.979 | -0.008 |
| L_Postcentral gyrus | 1.653 | 0.107 | 0.516 |  | 1.408 | 0.121 | 0.148 |
| L_Posterior cingulate | 0.201 | 0.842 | 0.062 |  | -0.433 | 0.668 | -0.132 |
| L_Precentral gyrus | 1.154 | 0.137 | 0.164 |  | 1.200 | 0.237 | 0.367 |
| L_Precuneus | 0.242 | 0.810 | 0.075 |  | 0.590 | 0.558 | 0.180 |
| L_Rostral anterior cingulate | 0.847 | 0.402 | 0.263 |  | -0.022 | 0.982 | -0.007 |
| L_Rostral middle frontal gyrus | 0.465 | 0.644 | 0.143 |  | 0.838 | 0.407 | 0.259 |
| L_Superior frontal gyrus | 0.519 | 0.606 | 0.160 |  | -0.156 | 0.877 | -0.048 |
| L_Superior parietal lobule | 0.888 | 0.380 | 0.277 |  | 0.902 | 0.373 | 0.274 |
| L_Superior temporal gyrus | 1.187 | 0.242 | 0.366 |  | 0.775 | 0.443 | 0.235 |
| L_Supramarginal gyrus | 0.215 | 0.831 | 0.066 |  | 0.767 | 0.448 | 0.233 |
| L_Frontal pole | -0.131 | 0.896 | -0.040 |  | 0.600 | 0.552 | 0.184 |
| L_Temporal pole | 1.259 | 0.132 | 0.118 |  | 0.175 | 0.862 | 0.054 |
| L_Transverse temporal cortex | 0.428 | 0.671 | 0.130 |  | -0.312 | 0.757 | -0.095 |
| L_Insula | 1.360 | 0.181 | 0.419 |  | 0.402 | 0.690 | 0.126 |
| R_Banks of the superior temporal sulcus | -0.117 | 0.908 | -0.036 |  | -0.075 | 0.941 | -0.023 |
| R_Caudal anterior cingulate | -1.213 | 0.232 | -0.373 |  | -2.128 | 0.040 | -0.652 |
| R_Caudal middle frontal | 0.526 | 0.602 | 0.164 |  | 0.889 | 0.381 | 0.280 |
| R_Cuneus | -0.451 | 0.654 | -0.137 |  | -0.226 | 0.823 | -0.071 |
| R_Entorhinal cortex | -0.137 | 0.891 | -0.042 |  | 0.566 | 0.575 | 0.176 |
| R_Fusiform gyrus | -1.168 | 0.251 | -0.366 |  | -0.043 | 0.966 | -0.013 |
| R_Inferior parietal lobule | -0.613 | 0.544 | -0.190 |  | 0.068 | 0.946 | 0.021 |
| R_Inferior temporal gyrus | 0.822 | 0.417 | 0.258 |  | 1.191 | 0.243 | 0.378 |
| R_Isthmus of the cingulate | 1.781 | 0.083 | 0.543 |  | 0.728 | 0.471 | 0.224 |
| R_Lateral occipital gyrus | -0.238 | 0.813 | -0.075 |  | -0.464 | 0.646 | -0.144 |
| R_Lateral orbitofrontal cortex | 1.017 | 0.150 | 0.121 |  | 1.486 | 0.117 | 0.166 |
| R_Lingual gyrus | 0.112 | 0.912 | 0.034 |  | 1.404 | 0.168 | 0.428 |
| R_Medial orbitofrontal cortex | 0.183 | 0.855 | 0.057 |  | 1.171 | 0.249 | 0.363 |
| R_Middle temporal gyrus | -0.499 | 0.621 | -0.157 |  | 0.067 | 0.947 | 0.021 |
| R_Parahippocampal gyrus | 1.464 | 0.151 | 0.452 |  | -0.481 | 0.635 | -0.142 |
| R_Paracentral gyrus | 0.073 | 0.942 | 0.022 |  | 0.015 | 0.988 | 0.005 |
| R_Pars opercularis | 1.402 | 0.169 | 0.431 |  | 1.851 | 0.072 | 0.567 |
| R_Pars orbitalis | 1.140 | 0.261 | 0.348 |  | -0.178 | 0.859 | -0.055 |
| R_Pars triangularis | 0.904 | 0.371 | 0.278 |  | 0.805 | 0.426 | 0.249 |
| R_Pericalcarine fissure | 1.413 | 0.166 | 0.433 |  | 0.729 | 0.470 | 0.223 |
| R_Postcentral gyrus | 1.670 | 0.103 | 0.514 |  | 1.626 | 0.112 | 0.502 |
| R_Posterior cingulate | 0.306 | 0.762 | 0.092 |  | 0.068 | 0.946 | 0.021 |
| R_Precentral gyrus | 0.913 | 0.367 | 0.282 |  | 1.319 | 0.195 | 0.412 |
| R_Precuneus | -0.771 | 0.446 | -0.238 |  | -0.029 | 0.977 | -0.009 |
| R_Rostral anterior cingulate | 1.190 | 0.243 | 0.376 |  | -0.499 | 0.621 | -0.153 |
| R_Rostral middle frontal gyrus | 1.485 | 0.146 | 0.461 |  | 1.091 | 0.282 | 0.337 |
| R_Superior frontal gyrus | 0.270 | 0.788 | 0.083 |  | 1.208 | 0.234 | 0.374 |
| R_Superior parietal lobule | 0.026 | 0.980 | 0.008 |  | 0.585 | 0.562 | 0.180 |
| R_Superior temporal gyrus | 0.220 | 0.827 | 0.069 |  | 0.708 | 0.483 | 0.216 |
| R_Supramarginal gyrus | 0.676 | 0.503 | 0.207 |  | 1.324 | 0.193 | 0.403 |
| R_Frontal pole | -0.257 | 0.798 | -0.080 |  | -0.282 | 0.779 | -0.087 |
| R_Temporal pole | 0.189 | 0.851 | 0.059 |  | -0.981 | 0.333 | -0.307 |
| R_Transverse temporal cortex | -0.128 | 0.899 | -0.039 |  | 0.456 | 0.651 | 0.139 |
| R_Insula | -0.235 | 0.816 | -0.073 |  | 0.675 | 0.504 | 0.210 |
| *Notes*. CSA, cortical surface area; CT, cortical thickness; L, left; R, right. | | | | | | | |

| **Table S4.** Group differences in nodal network metrics of SCNs based on CSA and CT at baseline and 3-year follow up (P-value after FDR correction) | | | | |
| --- | --- | --- | --- | --- |
| Baseline | | | | |
|  | Metrics | Hemisphere | Brain region | P-value |
| CSA | Nodal Degree | Left | Entorhinal cortex | 0.018 |
|  | Nodal Degree | Left | Superior temporal sulcus | 0.037 |
|  | Nodal Efficiency | Left | Entorhinal cortex | 0.006 |
|  | Nodal BC | Left | Lateral orbitofrontal cortex | 0.049 |
| CT | Nodal Degree | Right | Parahippocampal gyrus | 0.048 |
|  | Nodal Efficiency | Right | Isthmus cingulate cortex | 0.007 |
|  | Nodal BC | Left | Banks of the superior temporal sulcus | 0.035 |
|  | Nodal BC | Right | Lingual | 0.016 |
| 3-year follow-up | | | | |
|  | Metrics | Hemisphere | Brain region | P-value |
| CSA | Nodal Degree | Left | Entorhinal cortex | 0.01 |
|  | Nodal Efficiency | Left | Entorhinal cortex | 0.002 |
|  | Nodal Efficiency | Left | Parahippocampal gyrus | 0.044 |
|  | Nodal Efficiency | Left | Superior frontal gyrus | 0.028 |
|  | Nodal Efficiency | Left | Frontal pole | 0.019 |
|  | Nodal Efficiency | Left | Temporal pole | 0.018 |
|  | Nodal BC | Left | Lateral occipital cortex | 0.009 |
| CT | Nodal Efficiency | Left | Superior frontal gyrus | 0.034 |
|  | Nodal BC | Left | Superior frontal gyrus | 0.012 |
|  | Nodal BC | Right | Medial orbitofrontal cortex | 0.017 |
|  | Nodal BC | Right | Pars triangularis | 0.026 |
|  | Nodal BC | Right | Superior frontal gyrus | 0.034 |
|  | Nodal BC | Right | Temporal pole | 0.024 |
| *Notes*. SCNs, structural covariance networks; CSA, cortical surface area; CT, cortical thickness; FDR, false discovery rate; BC, betweenness centrality. | | | | |
